# Exhaled Volatile Organic Compounds Detection in Pneumonia Screening: A Comprehensive Meta-analysis

**DOI:** 10.1007/s00408-024-00737-8

**Published:** 2024-08-24

**Authors:** Juan He, Ran Zhong, Linlu Xue, Yixuan Wang, Yang Chen, Zihui Xiong, Ziya Yang, Sitong Chen, Wenhua Liang, Jianxing He

**Affiliations:** 1 Department of Thoracic Surgery and Oncology, National Center for Respiratory Health, Guangzhou Institute of Respiratory Health, the First Affiliated Hospital of Guangzhou Medical University, Guangzhou, 510120 China; 2 Nanshan School, Guangzhou Medical University, Jingxiu Road, Panyu District, Guangzhou, 511436 China; 3 The First Clinical School, Guangzhou Medical University, Jingxiu Road, Panyu District, Guangzhou, 511436 China; 4 ChromX Health Company Limited, Guangzhou, 510120 China; 5 Guangzhou Yuexiu Huanghuagang Street Community Health Service Center, Guangzhou, 510075 China

**Keywords:** Volatile organic compounds, Pneumonia, Breath, Diagnostic accuracy

## Abstract

**Background:**

Pneumonia is a common lower respiratory tract infection, and early diagnosis is crucial for timely treatment and improved prognosis. Traditional diagnostic methods for pneumonia, such as chest imaging and microbiological examinations, have certain limitations. Exhaled volatile organic compounds (VOCs) detection, as an emerging non-invasive diagnostic technique, has shown potential application value in pneumonia screening.

**Method:**

A systematic search was conducted on PubMed, Embase, Cochrane Library, and Web of Science, with the retrieval time up to March 2024. The inclusion criteria were diagnostic studies evaluating exhaled VOCs detection for the diagnosis of pneumonia, regardless of the trial design type. A meta-analysis was performed using a bivariate model for sensitivity and specificity.

**Results:**

A total of 14 diagnostic studies were included in this meta-analysis. The pooled results demonstrated that exhaled VOCs detection had a combined sensitivity of 0.94 (95% CI: 0.92–0.95) and a combined specificity of 0.83 (95% CI: 0.81–0.84) in pneumonia screening, with an area under the summary receiver operating characteristic (SROC) curve (AUC) of 0.96. The pooled diagnostic odds ratio (DOR) was 104.37 (95% CI: 27.93–390.03), and the pooled positive and negative likelihood ratios (LR) were 8.98 (95% CI: 3.88–20.80) and 0.11 (95% CI: 0.05–0.22), indicating a high diagnostic performance.

**Conclusion:**

This study highlights the potential of exhaled VOCs detection as an effective, non-invasive screening method for pneumonia, which could facilitate future diagnosis in pneumonia. Further high-quality, large-scale studies are required to confirm the clinical utility of exhaled VOCs detection in pneumonia screening. **Study registration**: PROSPERO, Review no. CRD42024520498.

**Supplementary Information:**

The online version contains supplementary material available at 10.1007/s00408-024-00737-8.

## Introduction

Pneumonia remains a significant global health burden, with approximately 10% of patients requiring intensive care unit (ICU) admission [1]. Despite extensive research efforts, early diagnosis of pneumonia remains challenging. Traditional diagnostic methods, such as chest X-rays, blood tests, and sputum cultures, have limitations, including suboptimal sensitivity, specificity, and prolonged detection times [2–4]. The coronavirus disease 2019 (COVID-19) pandemic has further highlighted the need for rapid and accurate diagnostic tools to contain the spread of the disease [5, 6]. Currently, reverse transcription-polymerase chain reaction (RT-PCR) is the primary method for diagnosing COVID-19 by detecting severe acute respiratory syndrome coronavirus 2 (SARS-CoV-2) RNA in biological samples [7, 8]. However, RT-PCR has several drawbacks, including longer detection times, the need for various biological reagents, and potential patient discomfort during sample collection, which may lead to false-negative results.

Volatile organic compounds (VOCs) in exhaled breath have emerged as a promising non-invasive diagnostic tool for various diseases, including pneumonia [9–11]. Physiological processes, such as gas exchange in the alveoli, can be disrupted during pneumonia, leading to alterations in the concentrations of VOCs in the breath [12]. Breath testing has been clinically employed to diagnose conditions such as Helicobacter pylori infections and asthma [13]. Recent studies have investigated the diagnostic potential of VOCs in respiratory diseases, including community-acquired pneumonia (CAP) and hospital-acquired pneumonia (HAP). This non-invasive, rapid, and convenient diagnostic approach facilitates individualized treatment, reduces antibiotic misuse, and minimizes hospital stay and cross-infection risks. As technology advances and clinical research deepens, VOCs testing is anticipated to become a crucial tool in diagnosing CAP, HAP, and ventilator-associated pneumonia (VAP), thereby enhancing diagnostic accuracy and treatment outcomes [14–18].

Although several studies have investigated the application of exhaled VOCs detection in the diagnosis of pneumonia, there is heterogeneity and inconsistency among the findings [19, 20]. This pooled-analysis aims to systematically evaluate the sensitivity, specificity, and diagnostic accuracy of exhaled VOCs detection in pneumonia diagnosis, providing reliable medical evidence.

### Methods

## Search and Study Selection

This pooled-analysis was conducted in accordance with PRISMA guidelines. Two reviewers searched the PubMed, EMBASE, Cochrane Library, and Web of Science databases for literature published up to March 1, 2024. The keywords used were “pneumonia,” “volatile organic compounds,” and “exhalation screening,” and the appropriate medical subject heading (MeSH) terms. A specific search strategy was employed for each database, and the protocol was registered with PROSPERO, Review no. CRD42024520498.

## Eligibility Criteria

The inclusion criteria for the controlled trials are: patients with pneumonia confirmed through pathological or nucleic acid testing, supported by imaging modalities (chest X-ray, HRCT) and microbiological tests (sputum culture, bronchoalveolar lavage); detection of VOCs in the exhalations of subjects; and clinical studies. Exclusion criteria include studies with small sample sizes; lack of a healthy control group; studies focusing solely on VOCs detection technology; studies on changes in VOCs before and after pneumonia treatment; studies that did not report detected VOCs results; and articles not written in English or unpublished.

## Data Extraction

In addition to basic information such as authors and publication dates, we extracted relevant information about the experimental and control groups from the articles. All data related to the target outcomes were recorded in a Microsoft Excel database. This includes the names of authors, year of publication, data on study subjects in both experimental and control groups, methods of VOCs detection, and the diagnostic performance of exhaled VOCs detection. Since nearly all articles didn’t provide the number of true positives (TP), false positives (FP), true negatives (TN), and false negatives (FN), these metrics were calculated in this paper using the sensitivity, specificity, and the number of subjects in the experimental and control groups provided in the literature.

## Data Analysis

We used a bivariate random-effects model to calculate pooled sensitivity, specificity, positive likelihood ratio (LR +), and negative likelihood ratio (LR-) with associated 95% confidence intervals (CIs). We additionally used a hierarchical summary receiver operating characteristic (SROC) curve with a 95% CI region to further assess the diagnostic properties of the tests. Heterogeneity was summarized with I^2^ statistics for the univariate analyses of sensitivity and specificity. The software used for the pooled-analysis included Microsoft Excel, Origin 2021, and Meta-DiSc 1.4.

## Assessment of Study Quality

The quality of the included studies was assessed using the Quality Assessment of Diagnostic Accuracy Studies (QUADAS-2) tool, which evaluates four key areas: patient selection, index tests, reference standards, and the flow and timing of the study [21]. A bias risk assessment was performed for each component, and the first three areas were also assessed for their clinical applicability. The results of this assessment were classified into three risk levels: low, high, and unclear.

## Results

### Study Characteristics

**Fig. 1:**
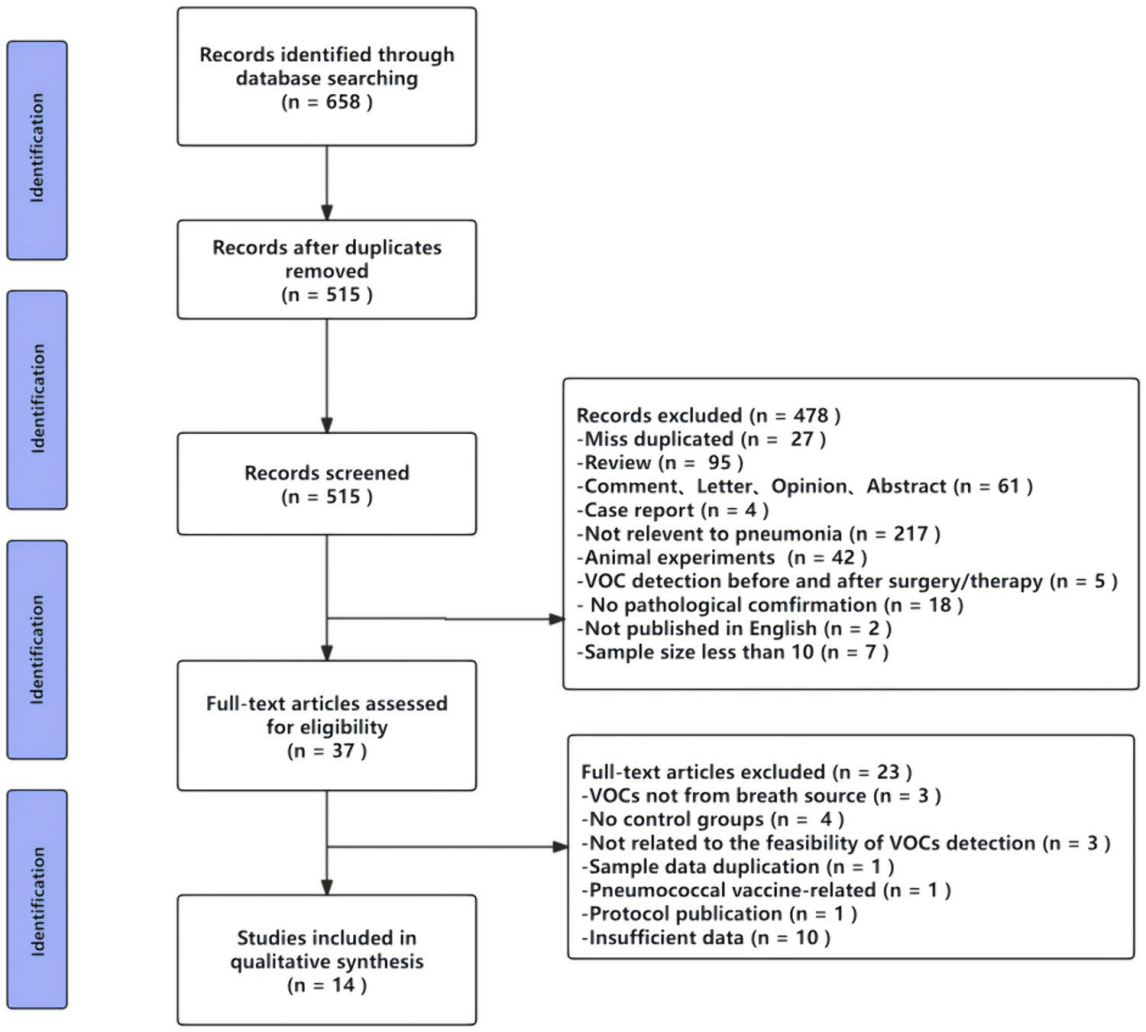
PRISMA flow chart illustrating the evidence search and study selection, PRlSMA, Preferred Reporting Items for Systematic Reviews and Meta-Analyses.

**Fig. 2:**
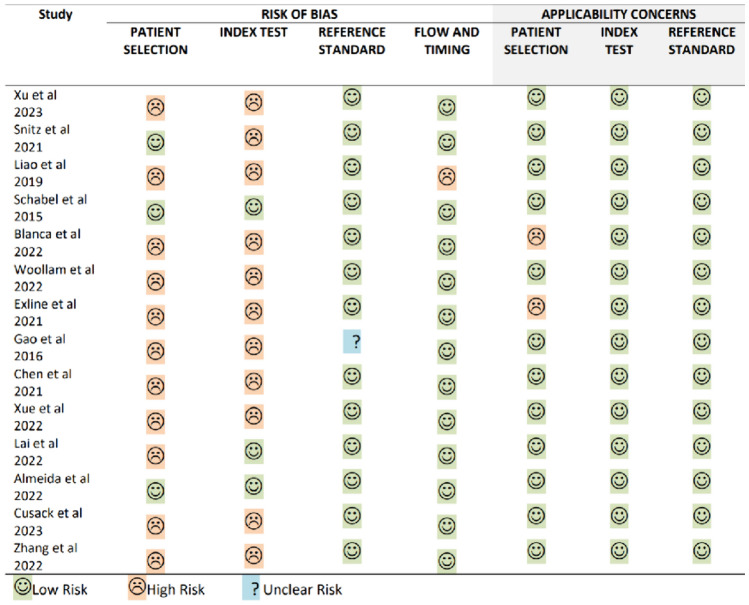
QUADAs-2 risk of bias assessment of forteen included studies, QUADAs, Quality Assessment of Diagnostic Accuracy Studies.

A preliminary identification of 658 studies was made using a specified search strategy (Fig. 1). After removing duplicates, 515 studies were considered for screening. Of these, 501 were excluded for not meeting the inclusion criteria, and a total of 14 studies were included. The included studies focus on the detection of VOCs for pneumonia diagnosis, involving a total of 690 pneumonia patients and 2129 control group participants (Table 1) [19, 22–34]. These studies originate from eight different countries, with a majority from China. The results of quality assessment were classified into three risk levels: low, high, and unclear. Thirteen studies were found to be of high or unclear risk of bias (Fig. 2).

**Table 1:** Baseline characteristics of included studies

Number	1	2	3	4	5	6	7	8	9	10	11	12	13	14
Author	Xu et al	Snitz et al	Liao et al	Schabel et al	Blanca et al	Woollam et al	Exline et al	Gao et al	Chen et al	Xue et al	Lai et al	Almeida et al	Cusack et al	Zhang et al
Year	2023	2021	2019	2015	2022	2022	2021	2016	2021	2022	2022	2022	2023	2022
Country	China	America	China	Netherlands	Mexico	India	America	China	China	China	China	Brazil	Canada	China
Vocs Detection method	PTR-MS	e-nose	e-nose	PTR-ToF–MS	e-nose	GC–MS	Multiplexed Nanomaterial-Based Sensor Array	GC–MS	GC-IMS	GC–MS	Nanomaterial-based hybrid sensor array	Terahertz	CRDS	HPPI-TOFMS
Statistical methods	Mann–Whitney U-test; stepwise discriminant analysis	LSTM deep-learning classifier algorithm	ENN; SVM	SVM;Chi-squared tests; t-tests	PCA;CDA;SVM	Mann–Whitney U-test; Linear regression analysis	Pearson Chisquare test; Student’s t.test; Wilcoxon Rank Sum analysis	PCA;PLS-DA; Mann–Whitney U-test	SVM;GBMs; RF	Mann–Whitney U-test; SVM;PCA	Artificial intelligence pattern recognition cloud algorithm	Student's unpaired t-test	SVM;Chi-squared tests; t-tests	RF;SVM;LR; XGB;KNN;DT
Collection methods	Intubation sampling	One-way disposable sampling valve	Intubation sampling	Tedlar bag	Metallized plastic bag	Tedlar bag	Tedlar bag	Tenax tube	ALTEF gas sample bags	Tedlar bag	Single-use disposable mouthpiece	Disposable Teratube	Tenax TA	polyether-ether-ketone sampling bag
Type Sample	VAP	COVID-19	VAP	VAP	COVID-19	COVID-19	COVID-19	VAP	COVID-19	COVID-19	COVID-19	COVID-19	COVID-19	COVID-19
Total	74	503	24	100	72	26	46	53	161	122	752	570	115	201
Pneumonia	42	476	12	32	42	14	23	33	74	65	108	70	53	95
Control	32	27	12	68	30	12	23	20	87	57	644	500	62	106
Pneumonia sample:														
Gender														
Male	25	252	10	26	28	10	14	21	33	31	69	41	36	64
Female	17	217	2	6	14	4	9	12	41	34	39	29	17	31
Age(mean ± SD / min–max)	65.2 ± 17.2	NA	70.17 ± 14.33	64 ± 12	38 ± 14	24–38	58–74	34–85	43.77 ± 13.69	27.78 ± 6.70	NA	38.6 ± 11.1	57.7 ± 17.1	29–49
Smoking														
Current smokers								8	18			4	1 (1.9)	13
Ex-smokers													11 (20.8)	
Never-smoked									56				39 (73.6)	82
Unknown														
Other Comorbidities														
Sepsis				11										
Gastrointestinal	3			4						3		38		
Cardiovascular	2			9			17	10		6		5	9	
Hematologic	6			3				6		2			34	7
Neurologic	12			4						12		78		
Respiratory	15			8			7			15		72	7	
Endocrine	2			0			12	8		2		3	21	
Other	2			4			16	3	24	2		41	2	4
Control Sample:														
Gender														
Male	16	15	7	44	7	5	11	12	34	29	303	233	32	44
Female	16	12	5	24	23	7	13	8	53	28	341	267	30	62
Age(mean ± SD/min–max)	71.8 ± 8.4	NA	44–87	60 ± 14.5	42.4 ± 10.8	23–57	54–72	44–77	39.67 ± 11.89	29.49 ± 6.59	NA	37.1 ± 12.5	57.5 ± 12.8	26–45
Smoking														
Current smokers								9	17			67	3 (4.8)	21
Ex-smokers													13 (21.0)	
Never-smoked									15				42 (67.7)	85
Unknown														
Other Comorbidities														
Sepsis				24										
Gastrointestinal	4			9						4		71		
Cardiovascular	9			13			21	10		9		43	1	
Hematologic	0			15				7		0			30	
Neurologic	12			6						12		260		
Respiratory	3			20			13			3		304	6	
Endocrine	3			0			5	8		3		12	14	
Other	1			5			28	6	14	1		53	1	2

^*^n is shown for categorical variables with percentage in parentheses. For continuous variables, median is shown with range in parentheses, *VAP* Ventilator-Associated Pneumonia, *COVID-19* Corona Virus Disease 2019, *SVM* Support Vector Machine, *RF* Random Forest, *ANN* Artificial Neural Networks, *LR* Logistic Regression *LOO* Leave-One-Out, *PCA* Principal Component Analysis, *ENN* Edited Nearest Neighbors, *LSTM* Long Short-Term Memory, *GC–MS* Gas Chromatography-Mass Spectrometry, *e-nose *electronic nose, *PTR-MS* Proton Transfer Reaction-Mass Spectrometry, *CRDS* Cavity Ring-down Spectroscopy, *PTR-ToF–MS *Fast Gas Chromatography-Proton Transfer Reaction-Mass Spectrometry, *GC-IMS* Gas Chromatography-Ion Mobility Spectrometry

**Fig. 3:**
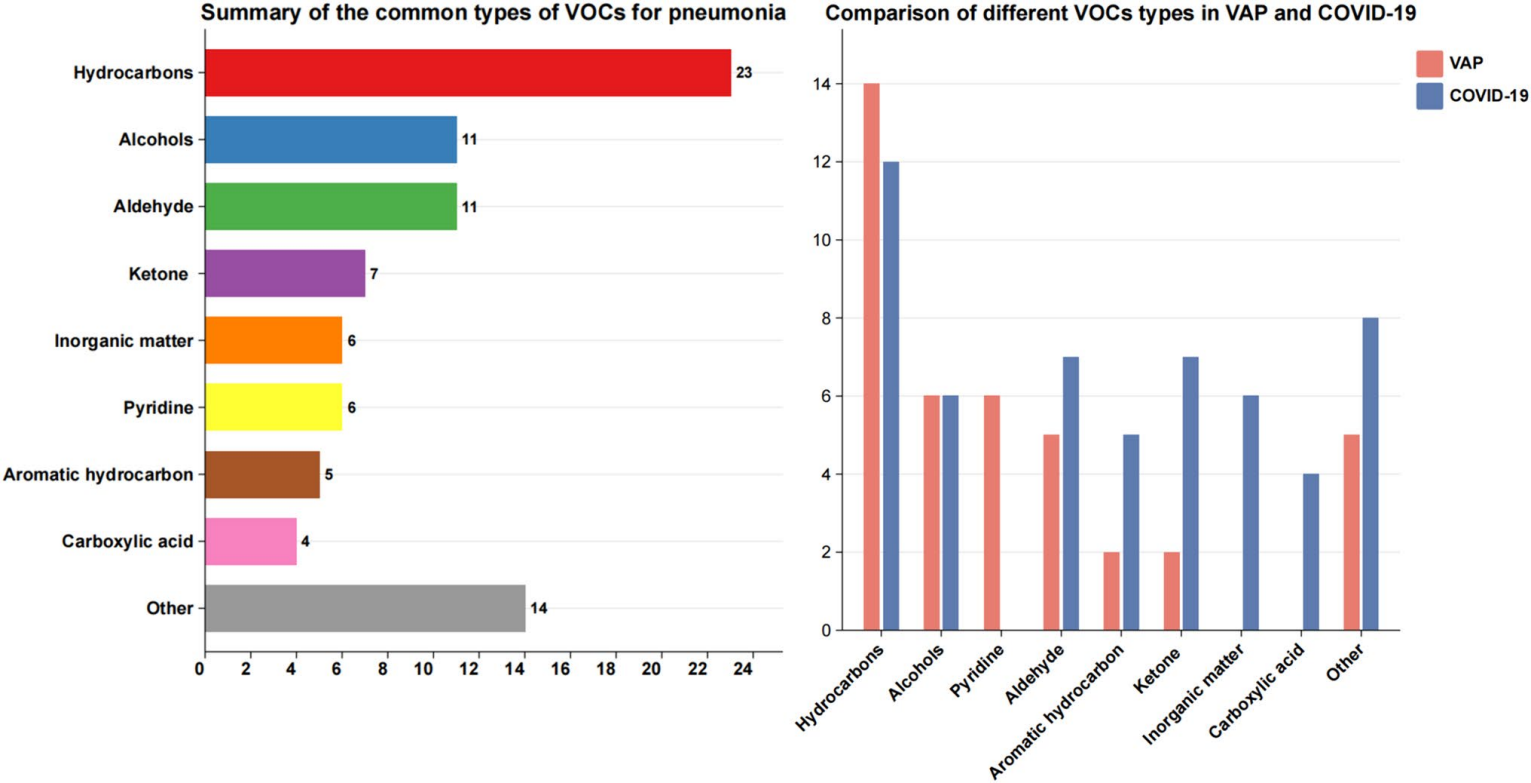
A summary of all common types of volatile organic compounds (different colors indicate different types of compounds) in the included studies, and comparison of each class in VAP and COVID-19. (VAP:Ventilator-associated pneumonia; COVID-19: SARS-CoV-2)

We cataloged all detected VOCs across the studies. The VOCs ranged from the most to the least prevalent types, including hydrocarbons, alcohols, aldehydes, ketones and terpenes, among others (Fig. 3). A total of 88 VOCs were detected in the breath of pneumonia patients across the included studies (Supplementary Table 1). However, among the 10 detection methods, only 14 VOCs were detected in two or more articles. Only 2 VOCs were detected in two or more articles in VAP, 7 VOCs in COVID-19, indicating low repeatability of VOCs detection across different studies (Supplementary Table 2 and 3).

## Pooled Results

**Fig. 4:**
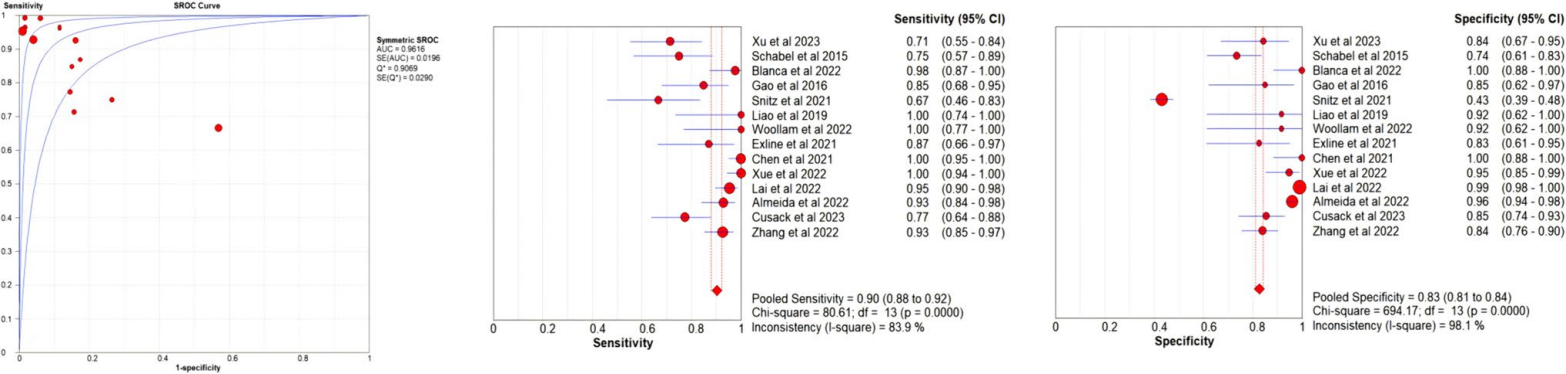
Plot of the summary receiver operating curve for the accuracy of exhaled VOCs detection in pneumonia screening (on the left). Each individual study is presented as a circle, and the 95% confidence region is presented as light blue line. And Descriptive forest plots of the sensitivity (on the middle) and specificity (on the right) of the exhaled VOCs detection in pneumonia screening

Based on the retrieved data, we estimated the accuracy of VOCs as a diagnostic tool for pneumonia. Fourteen diagnostic studies were included in this meta-analysis (Supplementary Table 4). The pooled results demonstrated that VOCs detection had a combined sensitivity of 0.94 and a combined specificity of 0.83 in pneumonia screening, with an area under the SROC curve of 0.96, indicating a high diagnostic performance. The study revealed that the sensitivity and specificity of exhaled VOCs for detecting pneumonia were 90% (95% CI: 88–92%) and 83% (95% CI: 81–84%), respectively. Significant heterogeneity was observed in the pooled sensitivity (I^2^ = 83.9%, P < 0.001) and pooled specificity (I^2^ = 98.1%, P < 0.001) (Fig. 4). The results of subgroup analysis showed that the sensitivity analysis for VAP and COVID-19 was 0.79 (95% CI: 71–86%) and 93% (95% CI: 90–95%), and the specificity analysis was 0.80 (95% CI: 72–86%) and 83% (95% CI: 81–85%), respectively (Table 2).

**Table 2:** Results of subgroup analyses of different types of pneumonia

Pneumonia type	TP	FP	FN	TN	Sensitivity	Specificity
VAP	94	27	25	105	0.79(0.71–0.86)	0.80(0.72–0.86)
COVID-19	529	331	42	1666	0.93(0.90–0.95)	0.83(0.81–0.85)
ALL-TYPE	623	358	67	1771	0.90(0.88–0.92)	0.83(0.81–0.84)

*TP *True Positives, *FP* False Positives, *TN* True Negatives, *FN* False Negatives

## Discussion

### Chemicals

The detected VOCs mainly include alkanes, alkenes, ketones, benzenes, aldehydes, and alcohols, with hexanal being the most common. Considering that these compounds have been mentioned in multiple studies, constructing a specific VOCs profile based on them could effectively improve the detection efficiency of pneumonia.

Some studies suggest that inflammation and oxidative stress processes in pneumonia patients may be important reasons for the production of certain VOCs. Pneumonia is an acute inflammatory disease caused by infection, and the inflammatory process produces a large number of reactive oxygen species (ROS), and other oxidative stress products [35, 36]. These ROS can attack unsaturated fatty acids on cell membranes, triggering lipid peroxidation reactions and producing a series of aldehydes and hydrocarbons [37]. Apart from inflammation and oxidative stress, pathogen metabolism may be another important source of specific VOCs in pneumonia patients. The main pathogens of pneumonia include bacteria, viruses, and fungi. These pathogens release some specific metabolic products during the infection process, which include some aldehydes, ketones, and hydrocarbons [38]. For example, pneumonia pathogens such as Streptococcus pneumoniae and Haemophilus influenzae produce some specific VOCs, such as acetaldehyde, acetone, and 2-butanone [39, 40].

In addition to local inflammatory responses, pneumonia may also cause systemic metabolic disorders, which also affect the VOCs profile. Pneumonia patients are often accompanied by systemic symptoms such as fever and hypoxemia, and these changes may affect the body’s energy metabolism and amino acid metabolism [41]. Metabolic disorders may lead to abnormal production or consumption of some VOCs, thereby altering the exhaled VOCs profile. It has been reported that diabetic patients produce acetone, intestinal bacterial activity produces methane and hydrogen, liver dysfunction produces sulfur-containing compounds (i.e., dimethyl sulfide, methyl mercaptan, and ethyl mercaptan), and nitrogen-containing compounds in renal impairment (i.e., dimethylamine, trimethylamine) [42].

## Pneumonia Types

Most of the included studies focused on specific types of pneumonia, such as COVID-19 or VAP, with few studies analyzing pneumonia as a broad category or defining subgroups based on pathological or radiological types. Xu et al. found that the concentration of exhaled acetaldehyde in VAP patients was correlated with the number of lower respiratory tract colonizing bacteria, confirming the contribution of lower respiratory tract pathogens to exhaled acetaldehyde in VAP patients. Although the ability to release acetaldehyde varies among different strains (including *P. aeruginosa*, *K. pneumoniae*, *A. baumannii*, *E. coli*, and *S. maltophiia*), the overall quantity of pathogens is higher, and the concentration of exhaled acetaldehyde is also higher. The sensitivity and specificity of screening VAP using acetaldehyde in exhaled breath were 71.4 and 84.4% [22]. Schabel et al. demonstrated that 12 VOCs could differentiate and explain ICU patients with and without VAP [19]. Gao et al. evaluated the discriminatory ability of 8 VOCs using ROC curves and suggested that these VOCs might originate from Pseudomonas aeruginosa VAP. Comparing the experimental group with the control group, the AUC was 0.88, and the corresponding optimal sensitivity and specificity both reached 85.0% [28]. Xue et al. showed that the VOCs profile in human breath changes even in the early stages of SARS-CoV-2 infection [30]. Almeida et al. indicated that co-infection with other respiratory viruses might not impair the accuracy of breath analysis methods [32]. Zhang et al. explained that VOCs-based COVID-19 detection methods are more sensitive to early infections with low viral loads. Autopsies also revealed that viral RNA in body tissues decreases in the middle and late stages of infection, but the levels of viral RNA in organ tissues remain persistently low [34].

Blanca et al. demonstrated that the damage caused by SARS-CoV-2 in different organs is associated with increased oxidative stress and hyperresponsiveness of the immune system induced by the cytokine storm, a process that can alter the overall cellular metabolism of the host [25]. Woollam et al. clustered COVID-19 subjects into two groups (COVID 1 and COVID 2). When using 16 VOCs, the discriminatory accuracy was 100% for all three groups (COVID 1, COVID 2, and control group), with nearly 50% of the differentially expressed VOCs identified as volatile terpenes/terpenoids (VTs) or their esters [26].

Therefore, there are some differences in the detection sensitivity between VAP and COVID-19. First, VAP pathogens are diverse, including bacteria and fungi, whereas COVID-19 is caused by a specific coronavirus, making it easier to develop targeted detection methods for the latter. Secondly, the diverse sources of VAP infections result in a more complex VOCs profile in exhaled breath, affecting detection sensitivity. Thirdly, VAP detection requires identifying metabolic products associated with multiple pathogens, which is technically complex, whereas COVID-19 detection is highly specialized, focusing on the single pathogen SARS-CoV-2, resulting in higher sensitivity.

## Collection Methods

Sample collection is an indispensable step in the detection of VOCs in breath but varies in current VOCs studies. Tedlar bags are the most commonly used collection method. Considering the low concentration of target VOCs in the samples and their sensitivity to interference, sample preparation often involves solid-phase microextraction (SPME), sorbent tubes, or thermal desorption (TD). In the study by Xu et al., the cohort comprised critically ill patients undergoing endotracheal intubation or tracheotomy, with sampling restricted to those with a stable expiratory phase to ensure sample stability [22]. Blanca et al. employed a method, where subjects performed three deep inhalations and exhalations into a metalized plastic bag pre-purified with ultra-pure nitrogen, enhancing sample stability [25]. Woollam et al. improved the procedure for collecting exhaled breath by connecting a ViroMax viral filter (viral filtration efficiency greater than 99.99%) to the Tedlar bag, which was proven to be effective in collecting exhaled breath with a increased carbon dioxide content [26]. Cusack et al. used Tenax tubes, which have stronger hydrophobicity compared to sampling bags and can better retain exhaled VOCs continuously [33]. Chen et al. used end-tidal breath samples for analysis. During each exhalation, the first half of the breath was exhaled into the surrounding environment, and then the remaining breath was exhaled into a gas bag, ensuring that only end-tidal breath, which better represents alveolar air in disease states and is less affected by the external environment, was collected [29].

## Detection Methods

Gas chromatography-mass spectrometry (GC–MS) is the gold standard for VOC detection and biomarker identification in human breath, known for its high accuracy but with high costs, time consumption, and limited portability. In contrast, the electronic-nose (e-nose) is a cheaper, faster, and portable option for pneumonia VOC detection, using a sensor array to detect resistance changes and generate specific response patterns for gases.

Some of the studies included in this research used other methods. The GS–IMS method combines GC with ion mobility spectrometry (IMS), offering rapid detection and swift operation [29]. Cavity ring-down spectroscopy (CRDS) is a highly sensitive laser spectroscopy that detects trace chemicals by measuring light absorption in a closed optical cavity, providing high specificity but lower sensitivity [33].

The High-Pressure-Photon-Ionization Time-Of-Flight Mass Spectrometry (HPPI–TOFMS) platform integrates a vacuum ultraviolet lamp HPPI ion source with an orthogonal acceleration TOF mass spectrometer. It has two ionization modes: soft-ionization and collision-induced dissociation, with accuracy similar to GC–MS. While it cannot measure VOC ion concentration precisely, the TOFMS signal correlates with VOC ion concentration, producing alternative indicators like compound mass spectral peaks [34].

Xu et al. used a self-made Proton-Transfer-Reaction Mass-Spectrometry (PTR–MS) instrument to measure VOCs, which has the advantages of fast detection speed, high sensitivity, and requires no sample pretreatment. However, PTR-MS only identifies ions based on m/z and has relatively weak qualitative ability. Therefore, Fast-Gas-Chromatography Proton-Transfer-Reaction Mass-Spectrometry (FGC–PTR–MS) was also used in the study to accurately identify characteristic ions [22]. Lai et al. employed the BreathPass™ device, which features a gas nanosensor system to capture breath fingerprints for biomarker analysis in volatile gases. VOCs patterns are analyzed using advanced AI algorithms in the cloud. The device includes a high-intensity ultraviolet system and an electrostatic filter in its sampling chamber to trap escaping viruses, enhancing the detection’s sensitivity and specificity (95.3 and 99.1%) [31].

## Statistical Methods

Traditional techniques like *t*-test, Wilcoxon signed-rank test, Mann–Whitney U-test, and principal component analysis (PCA) were used to explore linear associations between VOCs and pneumonia. However, these methods may miss complex nonlinear interactions between different VOCs. To address this, advanced machine learning techniques such as random forest (RF), support vector machine (SVM), and artificial neural networks (ANN) have been utilized in pneumonia diagnosis research [43, 44].

Xue et al. utilized the same dataset to construct and test four machine learning models, including SVM, ANN, KNN, and Logistic Regression (LR). The performance of the proposed SVM model was evaluated using ROC and compared with other machine learning models. The cross-validation optimized SVM model achieved a prediction accuracy of 97.3%, significantly higher than other machine learning models (ANN, KNN, and LR at 94.6%, 89.2%, and 91.9%) [22]. In addition to employing machine learning techniques, Blanca et al. introduced canonical PCA to order the data matrix and determine the misclassification levels between different sampling regions. Furthermore, by applying the leave-one-out method to variable prediction in the canonical space, this approach was able to maximize the overall classification success rate among the studied patient groups [25].

## Prospects for Future Research

This study highlights the potential of VOCs detection for diagnosing various types of pneumonia and identifies the standardization challenges in current VOC research, including inconsistent breath sampling, detection techniques, sampling methods, data analysis, and target VOC spectra selection. Given the emerging role of VOCs in diagnosing broader pulmonary diseases, establishing a standard VOC profile from a large, diverse sample of healthy individuals and developing comprehensive VOC profiles for all lung diseases are essential.

Future research should aim to improve VOCs detection accuracy, enhance instrument portability, and reduce detection time. Standardizing breath sampling, detection methods, data processing, and analysis techniques is crucial. Additionally, variables such as onset time, disease subgroups, smoking history, lifestyle, and comorbidities should be meticulously recorded and controlled.

## Limitation

Our study has several limitations. First, among the 14 included studies, most focused on specific types of pneumonia, such as COVID-19 or VAP, with few studies analyzing pneumonia as a broad disease category or determining subgroups based on pathological or imaging types. Additionally, the onset time and smoking history in participants was not consistently recorded. Only five of the included studies provided data on the smoking status. This lack of information may obscure the actual diagnostic efficacy of VOCs detection considering that these variables may affect the changes in VOCs profiles. Second, in the pooled-analysis of VOCs in pneumonia diagnosis, we observed heterogeneity in sensitivity and specificity. This variability arises from differences in patient populations (including types of pneumonia, age, gender, and other demographic characteristics), VOC detection methods (including sample collection, extraction, and processing steps), and analytical techniques and equipment (including detection techniques and data processing methods).

## Conclusion

Our study included the latest research findings on exhaled VOCs for the detection of pneumonia. This detection method has the advantages of being rapid, non-invasive, and having high patient compliance, showing broad prospects for clinical application. Certain compounds, such as alkanes, alcohols, and esters, were found to have a high correlation with pneumonia, indicating the promising potential of using specific VOCs in pneumonia detection. Given the limitations of current studies, factors affecting exhaled VOC profiles need further analysis and validation.

## Supplementary Information

Below is the link to the electronic supplementary material.

Supplementary file1 (XLSX 12 kb)

Supplementary file2 (XLSX 11 kb)

Supplementary file3 (XLSX 11 kb)

Supplementary file4 (XLSX 11 kb)

## Data Availability

No datasets were generated or analyzed during the current study.
